# Evaluation of the Antidiabetic and Antihyperlipidemic Activity of *Spondias purpurea* Seeds in a Diabetic Zebrafish Model

**DOI:** 10.3390/plants10071417

**Published:** 2021-07-12

**Authors:** Alethia Muñiz-Ramirez, Abraham Heriberto Garcia-Campoy, Rosa Martha Pérez Gutiérrez, Efrén Venancio Garcia Báez, José María Mota Flores

**Affiliations:** 1 CONACYT-IPICYT/CIIDZA, Camino a la Presa de San José 2055, Colonia, Lomas 4 Sección, San Luis Potosí CP 78216, Mexico; 2 Laboratorio de Investigación de Productos Naturales, Escuela Superior de Ingeniería Química e Industrias Extractivas, Instituto Politécnico Nacional, Ciudad de México CP 07708, Mexico; rmpg01@hotmail.com (R.M.P.G.); josemariamota@yahoo.com (J.M.M.F.); 3 Laboratorio de Química Supramolecular y Nanociencias, Instituto Politécnico Nacional, Acueducto S/N, Barrio la laguna Ticomán, Ciudad de México CP 07340, Mexico; efren1003@yahoo.com.mx

**Keywords:** *Spondias purpurea*, diabetes, zebrafish, antiglycation activity

## Abstract

Diabetes mellitus (DM) is a serious chronic degenerative disease characterized by high levels of glucose in the blood. It is associated with an absolute or relative deficiency in the production and/or action of insulin. Some of the complications associated with DM are heart disease, retinopathy, kidney disease, and neuropathy; therefore, new natural alternatives are being sought to control the disease. In this work, we evaluate the antidiabetic effect of *Spondias purpurea* seed methanol extract (CSM) in vitro and in a glucose-induced diabetic zebrafish model. CSM is capable of lowering blood glucose and cholesterol levels, as well as forming advanced glycation end-products, while not presenting toxic effects at the concentrations evaluated. These data show that CSM has a promising antidiabetic effect and may be useful in reducing some of the pathologies associated with diabetes mellitus.

## 1. Introduction

In recent decades, the impact and affection that diabetes mellitus (DM) has generated in the world has been alarming. In 2019, the number of people with diabetes was 463 million and is projected to reach 700 million by 2045 [1]. In Mexico, it is considered the second biggest cause of mortality [2]. DM is a chronic-degenerative disease related to elevated blood sugar levels; this causes damage to cells, organs, and systems [3]. Excess glucose in the blood results in the development of reactive oxygen species [4], inducing the proliferation of oxidative stress [5], which induces the formation of advanced glycation end products (AGEs) [6], the development of dyslipidemia [7], as well as a series of metabolic disorders linked to the disease. There are different types of diabetes, including type 1 diabetes (DM1), which is caused by insulin dependence. There is also type 2 diabetes (DM2), which is the most predominant and is related to the increase in glucose concentration and insulin resistance or sensitivity [8].

DM is complex and difficult to treat. There are different treatments that seek to regulate the concentration of glucose in the blood. Today, new alternatives capable of controlling the development of diabetic pathologies continue to be researched. Medicinal plants have traditionally been used as therapeutic systems [9], and ethnobotanical extracts have been analyzed for their richness in phytochemical compounds [10] due to their beneficial biological activities [11]. These bioactive agents have been isolated from different parts of plants (bark, lumen, branches, leaves, flowers, fruits, roots, and seeds) [12].

*S. purpurea*, better known as the Mexican plum, is a fruit tree belonging to the genus *Spondias*, which is made up of 17 species from the Anacardiaceae family [13]. Usually it grows on stony, sloping sandy or clayey soils with good surface drainage, although it has been reported that it can develop in soils with difficult drainage, which is why it is considered a robust species with high resistance to drought [14]. There are several reasons that justify its study and conservation, among which are: it is a native plant of Mexico, wild and cultivated specimens can be found, it is used as food by rural inhabitants, it adapts to low precipitation and high temperature conditions, and it produces at the dry time of the year when there are normally no fruits. It is a species that can subsist without cultivation and has the ability to survive without the presence of man [14].

*S. purpurea* is a deciduous tree with a height of 3 to 8 m, although it can measure up to 15 m [15]. Its endocarp is 0.5 to 0.75 cm long, it is large and fibrous, and inside it contains from 1 to 5 seeds. Its fruit is an ovoid drupe of red, orange, yellow, and green color with dimensions of 1.5 cm wide and 3 cm long [16]; it contains a yellow, juicy and bittersweet mesocarp [17] which is consumed fresh or processed (dehydrated, pickled or in brine). It is also used to make jellies and beverages, among other products [18]. Various studies have been carried out to determine the chemical composition [19] of the fruit [20], the rind [21], and the leaf [22], finding phenolic compounds, flavonoids [23], tannins [21], polysaccharides, essential oils, triterpenes saponins, sterols [24], and amino acids [25]. In addition, its antioxidant [16], antimicrobial [26], antifungal [27], antiulcer [28], photoprotective [23], and anti-glycation potential has been evaluated [29].

In recent years, interest has increased in developing reproducible animal models to evaluate molecules for the treatment of various diseases [30]. An example of this is the zebrafish model, which has been widely used for in vivo studies that include diabetes, cardiovascular disease, oxidative stress, melanoma, the immune system, and cancer [31]. The zebrafish (*Danio rerio*) is an emerging model of great importance in biomedicine [32]; in the last 60 years, it has allowed the identification, study, and evaluation of drugs, bioactives, natural extracts, or harmful substances capable of inducing therapeutic action or toxic to living beings [33]. This organism has approximately a 70% physiological and genetic similarity with humans [34], which has motivated its use in the development of research related to metabolic diseases [35] such as diabetes mellitus [36]. Zebrafish have the capacity to develop diabetes mellitus and its complications [37]. This condition can be induced by exposing the fish to a medium with a high glucose concentration [38]. The objective of this study is to evaluate the effect of the methanol extract from *S. purpurea* (CSM) seeds on the regulation of blood glucose, triglyceride, and cholesterol levels, as well as to determine whether the extract is capable of inhibiting the protein glycation reaction using a glucose-induced diabetic zebrafish model.

## 2. Results

### 2.1. Inhibition Tests for the Formation of Advanced Glycation End Products In Vitro

#### 2.1.1. Glycation of Bovine Albumin

**Figure 1:**
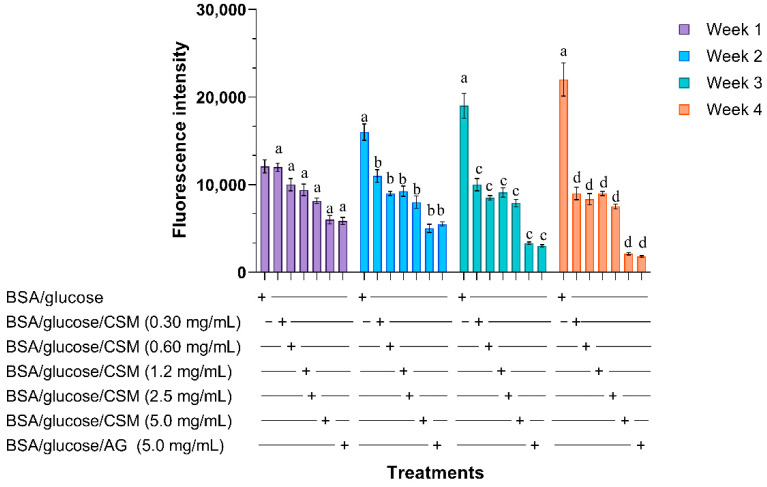
Effects of CSM extract on formation of fluorescent advanced glycation end products (AGEs) in BSA incubated with glucose. Each value represents the mean ± SE. ^a^ *p* ≤ 0.05 when compared to BSA/glucose at week one; ^b^ *p* ≤ 0.05 when compared to BSA/glucose at week two; ^c^ *p* ≤ 0.05 when compared to BSA/glucose at week three; ^d^ *p* ≤ 0.05 when compared to BSA/glucose at week four. Different letters indicate significant differences between the weeks evaluated. CSM; methanol extract from the seed of *S. purpurea*, AG; aminoguanidine.

The intensity in the fluorescence indicates the formation of AGEs. The fluorescence in the BSA/glucose system during 1, 2, 3 and 4 weeks of incubation is shown in Figure 1. A concentration-dependent decrease in fluorescence intensity is observed in the samples incubated with BSA/glucose/CSM. The concentration of 5 mg/mL at 4 weeks of incubation was the one that showed a greater decrease (90.45%) compared to glycated BSA over the same time interval. The BSA/glucose/AG system used as a control showed a decrease of 91.8%.

#### 2.1.2. Determination of Fructosamine

**Figure 2:**
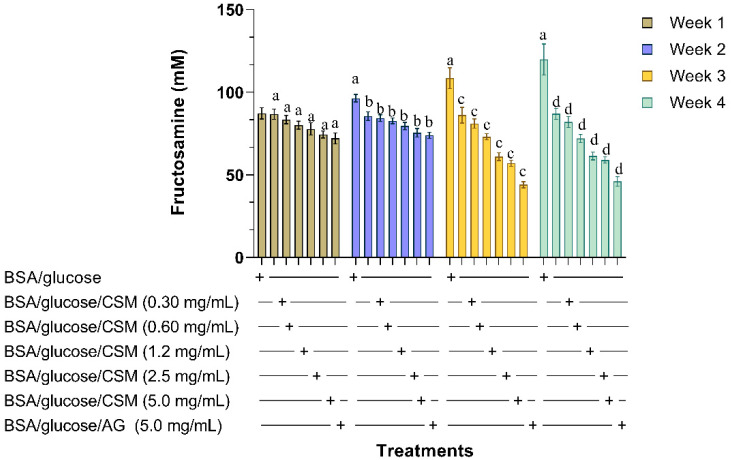
Effect of CSM on fructosamine levels during four weeks of incubation using the BSA/glucose system. Results are expressed as mean ± SE. ^a^ *p* ≤ 0.05 when compared to BSA/glucose at week one; ^b^ *p* ≤ 0.05 when compared to BSA/glucose at week two; ^c^ *p* ≤ 0.05 when compared to BSA/glucose at week three; ^d^ *p* ≤ 0.05 when compared to BSA/glucose at week four. Different letters indicate significant differences between the weeks evaluated. CSM; methanol extract from the seed of *S. purpurea*, AG; aminoguanidine.

Fructosamines are precursors to the formation of AGEs, therefore it is important to reduce their formation. In Figure 2, the effect of CSM at different concentrations on fructosamine levels can be observed during 1, 2, 3, and 4 weeks of incubation. A significant increase in glycated BSA was observed during the duration of the experiment. The BSA/glucose/CSM system at different concentrations decreased fructosamine levels over the weeks, with the concentration of 5 mg/mL showing a value of 59.85 mM in the fourth week of incubation compared to glycated BSA, which showed values of 119.6 mM over the same time interval.

#### 2.1.3. Determination of Nɛ-(carboxymethyl) Lysine

**Figure 3:**
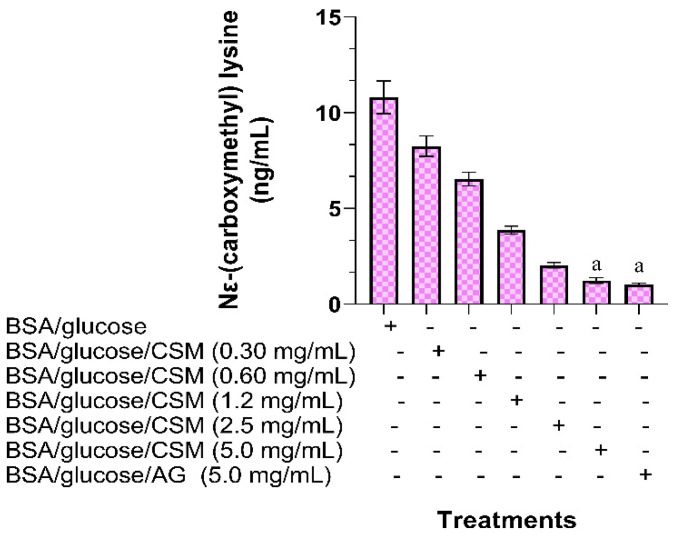
Effect of CSM on the inhibition of Nε-(carboxymethyl) lysine (CML) after four weeks of incubation. Each value represents the mean ± SE. ^a^ *p* ≤ 0.05 when compared to BSA/glucose. The letters indicate significant differences between the different treatments. CSM; methanol extract from the seed of *S. purpurea*, AG; aminoguanidine.

CML is a non-fluorescent type of AGE; therefore, it is important to quantify its formation. Figure 3 shows the effect of CSM on the inhibition of Nε-(carboxymethyl) lysine (CML) after four weeks of incubation. It can be seen that the decrease in the concentration of CML in the BSA/glucose/CSM system is dependent on the concentration. The greatest decrease is observed when using a concentration of 5 mg/mL of CSM (1.3 ng/mL). In comparison, glycated BSA presented a CML concentration of 10.8 ng/mL over the same time interval.

#### 2.1.4. BSA-Methylglyoxal Assay

**Figure 4:**
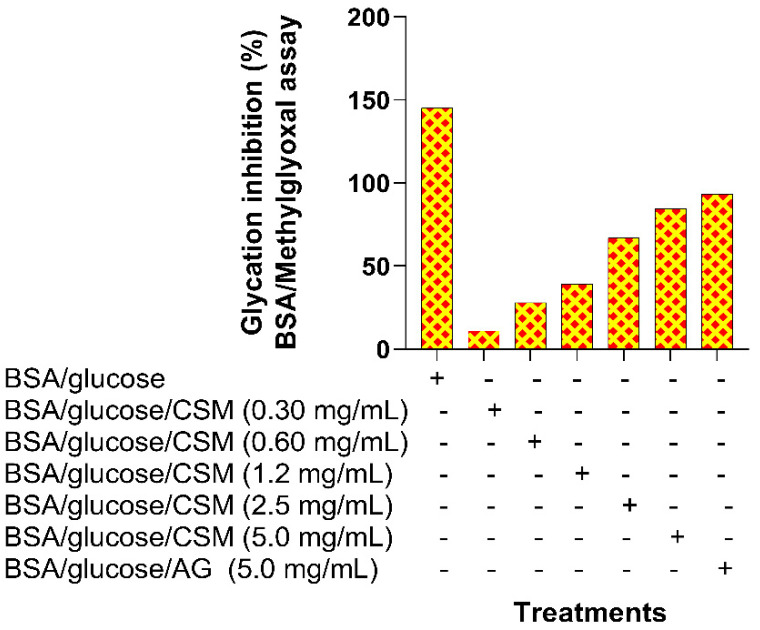
CSM inhibitory activity in the BSA/methylglyoxal model. Values are expressed as a percentage of glycation inhibition. CSM; methanol extract from the seed of *S. purpurea*. AG; aminoguanidine.

Methylglyoxal is a precursor in the formation of AGEs, and reducing their formation reduces the formation of these compounds. In Figure 4, the percentage of inhibition in the formation of methylglyoxal can be observed when using CSM at different concentrations. The highest percentage of inhibition was obtained in the BSA/glucose/CSM system at a concentration of 5 mg/mL, showing an inhibition of 85.3% compared to the BSA/glucose/AG system, which showed an inhibition of 93%.

### 2.2. In Vivo Experiments

#### 2.2.1. Toxicity Test of the Methanolic Extract of *Spondias purpurea*

The zebrafish has been used as an experimental model due to the great physiological similarity that it presents with mammals, in addition to having a small body size, low cost, and large clutch size [39]. Therefore, this model presents significant potential for pharmacological and toxicological studies. In the toxicological test, CSM did not show a toxic effect at the evaluated concentrations, and there were no significant differences in survival rates between treatment groups (*p* ≤ 0.05). Zebrafish survival rates in the CSM-treated groups were 100% at the end of the trial (96 h), compared with the group treated with 3,4-dichloroaniline, which presented 60% and 40% survival rates at 24 and 48 h, respectively. Regarding the results obtained, there is 99.9% confidence that the LC_50_ is greater than 100 mg/L [40].

#### 2.2.2. Hyperglycemia Induction

**Figure 5:**
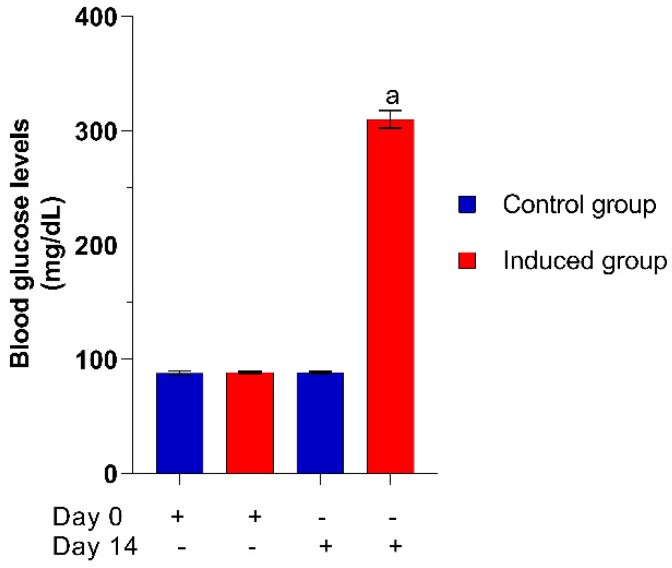
Elevation of blood glucose levels in zebrafish. Each value represents the mean ± SE. ^a^ *p* ≤ 0.05 when compared to normoglycemic control group. The letter indicates significant difference.

The induction of diabetes in zebrafish by means of 111 mM glucose is shown in Figure 5. At the beginning of the test, the fish showed blood glucose values of 88 mg/dL; on day 14 the fish treated with glucose showed blood glucose values of 310 mg/dL compared to the control group (water without glucose), which maintained glucose levels at 88 mg/dL.

#### 2.2.3. Effect of CSM in Blood Glucose Levels

**Figure 6:**
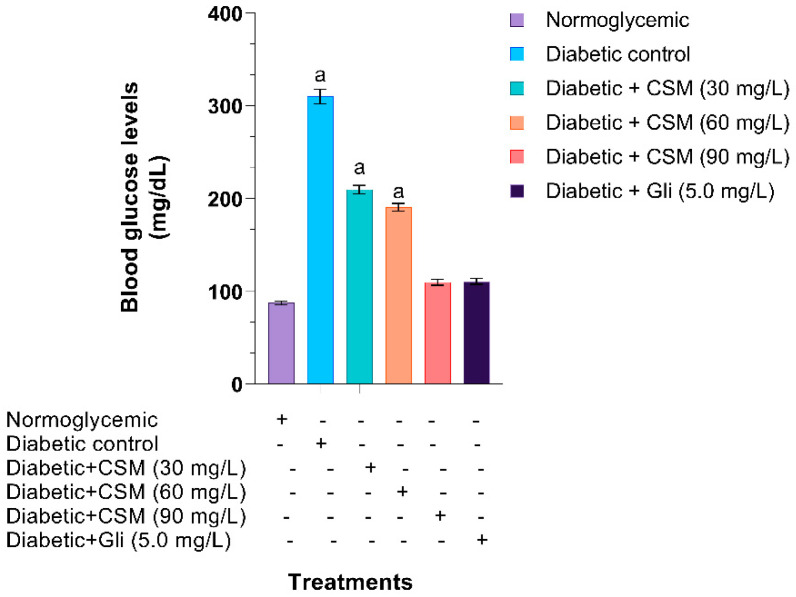
Effect of CSM on blood glucose levels of glucose-induced diabetic zebrafish. Each value represents the mean ± SE. ^a^ *p* ≤ 0.05 when compared to normoglycemic control group. The letter indicate significant differences between the different treatments. CSM; methanol extract from the seed of *S. purpurea*, Gli; glibenclamide.

Figure 6 shows the blood glucose levels of diabetic zebra fish treated with CSM. When administering the CMS extract, a concentration-dependent decrease in blood glucose was observed. The group treated with CSM (90 mg/L) and the one treated with glibenclamide showed blood glucose values of 110 mg/dL, while the normoglycemic group showed values of 80 mg/dL, compared to the diabetic group, which showed values of 310 mg/dL.

#### 2.2.4. Effect of CSM in Triglyceride and Cholesterol Levels

**Figure 7:**
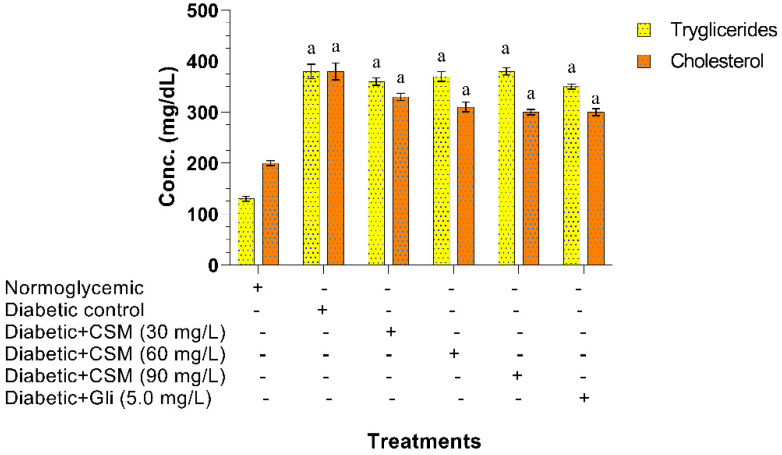
Effect of CSM on glucose-induced zebrafish triglycerides and cholesterol levels at the end of the experimental period (14 days). Data are expressed as the mean ± SE. ^a^ *p* ≤ 0.05. when compared to normoglycemic control group. The letters indicate significant differences between the different treatments. CSM; methanol extract from the seed of *S. purpurea*, Gli; glibenclamide.

The effect of CSM on triglyceride and cholesterol levels is shown in Figure 7. At the end of the experimental period, it was observed that the administration of CSM did not significantly decrease triglyceride levels. Regarding cholesterol levels, a significant decrease was observed (^a^ *p* ≤ 0.05), the concentration of 90 mg/L of CSM being that which presented a greater effect with values of 300 mg/dL compared to the diabetic group, which showed values of 380 mg/dL, while the normoglycemic group presented values of 200 mg/dL.

#### 2.2.5. Effect of CSM in the Inhibition of AGEs In Vivo

**Figure 8:**
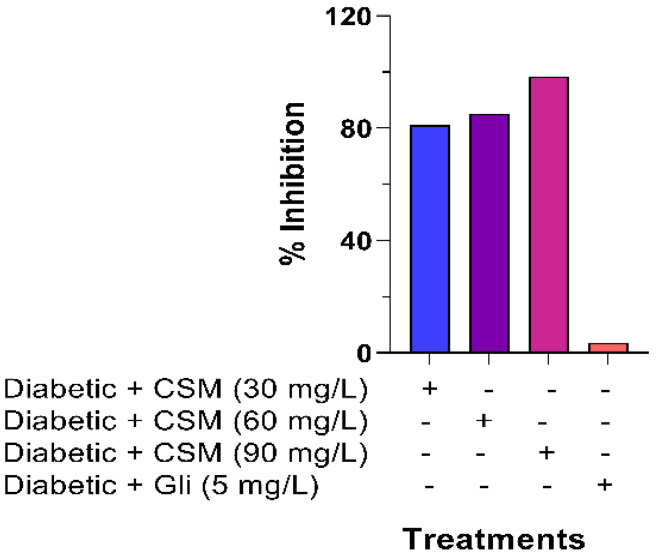
Effect of CSM on AGEs formation at the end of the experimental period (14 days). Values are expressed as a percentage of glycation inhibition. CSM; methanol extract from the seed of *S. purpurea*, Gli; glibenclamide.

The inhibition percentages in the formation of AGEs when administering CSM are shown in Figure 8. At the end of the experiment period, it was observed that all the evaluated concentrations of CSM showed a significant inhibition in the formation of AGEs. The concentration of 90 mg/dL of CSM showed the highest percentage of inhibition (98.5%).

## 3. Discussion

Zebrafish are an ideal experimental model to mimic the human pathological condition, since they have 70% of human genes that encode proteins and 70% to 84% of genes related to human diseases [30,41]. The zebrafish has a short maturation period, easy reproduction, good regeneration capacity, its development stages can be characterized, low maintenance cost, high productivity, fewer ethical restrictions, etc. This makes the zebrafish an ideal model for drug development and toxicity testing [42,43].

As many cellular processes are highly conserved between zebrafish and humans, as well as the organization of the genome and the pathways involved in controlling signal transduction, Zebrafish models have been developed to cover a wide range of human diseases [44].

The study of glucose metabolism is a good illustration of the metabolic models in zebrafish [45]. Studies indicate that zebrafish regulate glucose metabolism through the same enzymes and pathways as mice and humans. For example, high levels of glucose in zebrafish stimulate insulin expression [46] and negatively regulate gluconeogenesis [47]. Adult zebrafish, like mammals, have been documented to be sensitive to antidiabetic drugs that reduce glucose levels in blood [45], which further illustrates the physiological preservation of glucose regulation [48]. As in mammals, the pancreas of the zebrafish is made up of two types of tissues; exocrine and endocrine [49], which are responsible for the regulation of glucose metabolism through the secretion of insulin, somatostatin, and glucagon directly into the bloodstream [47]. Zebrafish also regulate the expression of phosphoenolpyruvate and carboxykinase (the enzyme that catalyzes one of the key stages of gluconeogenesis) by glucagon and insulin, like mammals [45]. The specification and morphogenesis of the pancreas of zebrafish allows this organism to be used as a physiological model of pancreatic function, as well as a model to study diabetes mellitus and its complications.

Adult zebrafish readily absorb water molecules due to their ability to regulate their internal water and total solute concentrations [50]. This is an osmoregulation strategy that involves continuous water gain because of a higher internal salt concentration compared to its freshwater environment. The constant entry of water results in the absorption of molecules from their environment. It is for this reason that a hyperglycemia model was proposed by immersing the zebrafish in a glucose solution, which is capable of generating a diabetic condition, as well as diabetic retinopathy similar to that of humans [44,51]. A diabetic condition is persistent after a period of glucose withdrawal, and zebrafish have been reported to be sensitive to antidiabetic medications [52].

In zebrafish, glucose uptake/consumption occurs through a glucose transporter called GLUT, which is expressed in the gills (GLUT 1–3, 6, 8, and 10–13) and intestine (GLUT 5 and 9) [53]. Our results show that exposure to a 111 mM glucose solution promotes an elevation in blood glucose levels in zebrafish, as reported by Gleeson et al., 2007 [54], and in turn, the glycosylation of protein is promoted in the eyes. Our results show that CSM can counteract the hyperglycemic state most likely by stimulating pancreatic islet cells, which causes an increase in insulin secretion. Furthermore, it has been reported that the effect of natural products compared to high blood glucose levels is due to their antioxidant effects [55,56]. However, studies are required to determine the action mechanism of CSM in lowering blood glucose.

The use of anesthetics such as MS-222 has been reported to affect blood glucose levels. MS-222 blocks nerve ion channels and may directly affect the ion channels of β cells, and therefore insulin secretion. Anesthetics generally alter glucose levels [57], and some have been shown to alter insulin secretion by acting directly on the channels of β cells. For example, tetracaine alters the absorption or Ca^2+^ flow of β cells depending on the dose, and isoflurane decreases the secretion of insulin from β cells by opening potassium channels that are sensitive to ATP [58,59]. Therefore, for studies involving glucose metabolism, choosing the right anesthetic is crucial. In this regard, we used cold water as anesthesia in this study, which has been reported to help keep blood glucose levels more stable at the time of reading [60].

Diseases such as neuropathy, atherosclerosis, retinopathy, cataracts, and diabetic nephropathy are promoted by AGEs [61]. The formation of reactive oxygen species (ROS) is one of the suggested mechanisms of damage caused by AGEs, specifically hydrogen peroxide and superoxide released by AGEs [62]. The oxidation of sugars and fructosamines are associated with the formation of free radicals. The protein non-enzymatic glycation reaction has been shown to increase free radical production 50-fold compared to non-glycated proteins [63]. The process begins with the production of superoxide radicals due to the autoxidation of proteins and sugars that bind to fructosamines, followed by the dismutation of superoxide into hydrogen peroxide with the subsequent generation of hydroxyl radicals. The above can cause a specific attack on proteins resulting in lipid peroxidation and protein damage [61].

The formation of AGEs can be detected in various proteins such as in the α- crystalline structure of the ocular lens or in the collagen of connective tissue; these proteins are exposed to glycation throughout their useful life. The sight impairment due to the opacity of the lens (cataracts) is one of the most common affections of diabetes derived from the formation of AGES [64].

A phytochemical sieve was made to CSM, which showed the presence of tannins, flavonoids, phenols, and glycosides. These compounds have been reported to act as antioxidants, helping to reduce the formation of free radicals and provide protection against various diseases such as diabetes mellitus [29]. Our results show that the CSM extract inhibits the formation of AGE in vitro and in vivo, probably due to the presence of these metabolites and their antioxidant effect, which helps to reduce the formation of ROS. The results obtained are comparable with those obtained when studying the hexane extract of *S. purpurea*, where a significant decrease in the formation of AGEs, CML, and fructosamine was observed [29]. It has been reported that the effects of most AGE inhibitors can be mainly attributed to their chelating capacity, since they could inhibit the autoxidation reactions that accompany glycation [65]. Therefore, discovering new inhibitors of the formation of AGEs provides a therapeutic option to prevent or treat some of the complications associated with diabetes mellitus.

Patients with type 2 diabetes present a group of lipid abnormalities that are associated with the accumulation of cholesterol and fatty acids in pancreatic β cells, which can contribute to the degeneration of pancreatic islets [65]. Excess cholesterol affects insulin release stimulated by glucose located in the endoplasmic reticulum, the mitochondria, and the cell membrane [66]. Our results indicate that CSM decreases cholesterol levels, which was reflected in a decrease in blood glucose levels. This could be due to the fact that CSM prevented the accumulation of cholesterol in the endoplasmic reticulum, favoring the release of insulin [67,68]. However, studies are required to determine the specific route by which CSM acts on cholesterol levels.

Both the fish and the zebrafish embryo are used to determine the acute toxicity of various compounds (including heavy metals, pesticides, and numerous compounds that cause environmental contamination). However, a similar approach can be used for the detection of acute toxicity in drugs and new therapeutic compounds [69]. Our results indicate that CSM does not present toxic effects at the concentrations evaluated, supporting the biocompatible nature of the extract. The zebrafish (*Danio rerio*) is firmly recognized as a powerful research model for many areas of biology and medicine. Zebrafish are especially valuable for drug discovery, as they represent a model organism to demonstrate the efficacy and toxicity of a new treatment before using more expensive mammalian models [45].

## 4. Materials and Methods

### 4.1. Raw Material Conditioning and Extract Preparation

The fruit of *S. purpurea* was collected in the State of Mexico (Cuautla), in April 2018. The seed was extracted from the fruit, dried in an air stream stove, and later the particle size was reduced in a manual mill. Two kilograms of dry and ground seed were extracted using 4 l of methanol by maceration. The extract obtained (CSM) was filtered and fully dried on a rotary evaporator to completely remove the solvent.

### 4.2. In Vitro Experiments

#### 4.2.1. In Vitro Glycation of Bovine Albumin

DMSO was added to the CSM extract to dissolve it, followed by the addition of 10 mg/mL of BSA, a phosphate buffer at pH 7.4, 1.1 M glucose, and finally sodium azide (0.2%). The previously prepared solution was incubated for 1, 2, 3, and 4 weeks at 37 °C. The glycation reaction was evaluated at an emission wavelength of 460 nm and excitation of 355 nm. Aminoguanidine (AG) was used as a positive control [70].

#### 4.2.2. Fructosamine Concentration

After incubation (1, 2, 3 and 4 weeks), the fructosamine concentration was evaluated using the nitroblue tetrazolium (NTB) assay [71]. 10 μL of glycated 0.1 M BSA and 0.1 M of carbonate buffer were added to 90 μL of 0.5 mM NTB at a pH of 10.4. The solution was shaken and incubated at 37 °C. After 10 to 15 min, absorbance was read at 530 nm. The concentration of fructosamine was calculated using the standard (1-deoxy-1-morpholino-fructose (1DMF)).

#### 4.2.3. BSA-Methylglyoxal Assay

To perform this test, 1 mL of catechin (1.5 mg/mL), sodium phosphate buffer (50 mM, pH 7.4), sodium azide (0.02%) and methylglyoxal (60 mM, 1 mL) were added to different CSM concentrations (0.30, 0.60, 1.2, 2.5 and 5 mg/mL) and incubated at 37 °C for 2 h. Subsequently, BSA (30 mg/mL, 1 mL) was added to the above mixture and incubated for six days at 37 °C. Phosphate buffer (1 mL) was used as a positive control. Aminoguanidine was also used as a positive control (final concentration 10 mM). After the incubation time, the samples were read at 380 nm of emission and 380 nm of emission [72]. The following equation was used to calculate the percentage of inhibition of AGEs:

$$\mathrm{percentage}\;\mathrm{inhibition}=1-\left(\frac{\mathrm{fluorescent}\;\mathrm{intensity}\;\mathrm{with}\;\mathrm{inhibitor}}{\mathrm{fluorescent}\;\mathrm{intensity}\;\mathrm{without}\;\mathrm{inhibitor}}\right)\times 100$$

#### 4.2.4. Determination of Nɛ-(carboxymethyl) Lysine

After 4 weeks of incubation, the concentration of Nε-(carboxymethyl) lysine (CML), was evaluated using the enzyme linked immunosorbant assay (ELISA) kit. The CML-BSA standard curve of the kit was used to calculate the CML concentration.

### 4.3. In Vivo Experiments

#### 4.3.1. Conditioning of Adult Zebrafish

Adult zebrafish with a body length of 2.5–3.0 cm were used; these were acclimatized for 15 days in 40 L tanks at 25 ± 2 °C, maintaining constant filtration and aeration. The fish were kept in a photoperiod of 14 h of light, 10 h of darkness daily and fed twice a day with commercial food (Azoo Plus, for tropical fish).

#### 4.3.2. Toxicity Test of the Methanolic Extract of *Spondias purpurea*

The toxicity test was carried out by following the recommendations of Organization for Economic Cooperation and Development (OECD) [40]. Different batches of 10 fish each were placed in tanks with 6 L of water at 23 ± 2 °C and constant aeration. The batches were formed as follows: batch 1: water (control); batch 2: water + 30 mg/L CSM, batch 3: water + 60 mg/L CSM; batch 4: water + 100 mg/L CSM; batch 5: water + 5 mg/L 3,4 dichloroaniline (positive control). The CSM extract and 3,4 dichloroaniline were renewed every 24 h to maintain a constant concentration. The above conditions were maintained for a period of 96 h; the fish from the different batches did not feed during this period. During the development of the test, the fish were constantly observed to assess their way of swimming, the movement of their gills, or the presence of deaths.

#### 4.3.3. Hyperglycemia Induction

Five batches of 25 fish were placed in 20 L fish tanks for a period of 14 days; the tanks contained a 111 mM glucose solution. The glucose solutions were interchanged every third day to avoid contamination by microorganisms. The organisms were continuously monitored for signs of stress, including excessive gill movement, as well as difficulty swimming. The control group were kept in fish tanks without the addition of glucose. During the development of the test, the fish were fed twice a day [54]. At the end of the induction of diabetes, fish were taken at random from each batch to corroborate their diabetic status, which was maintained throughout the experiment.

#### 4.3.4. Administration of the Methanol Extract of *Spondias purpurea*

##### Experimental Design

Twenty zebrafish in a diabetic state were taken at random to form the different groups and placed in 15 L tanks. The methanol extract from the *S. purpurea* seed (CSM) was dissolved and added to the tanks at different concentrations (30, 60 and 90 mg/L). Glibenclamide (5 mg/L) was used as positive control and the negative control received no treatment. The different treatments were administered daily for a period of 14 days. At the end of the experimental period, the fish were slaughtered.

- group 1: normoglycemic fish (without treatment administration).
- group 2 (negative control): glucose-induced diabetic fish (without treatment administration).
- group 3: glucose-induced diabetic fish administered with 30 mg/L CSM.
- group 4: glucose-induced diabetic fish administered with 60 mg/L CSM.
- group 5: glucose-induced diabetic fish administered with 90 mg/L CSM.
- group 6 (positive control): glucose-induced diabetic fish administered with 5 mg/L glibenclamide.

#### 4.3.5. Anesthesia and Sacrifice

The fish were placed in cold water at 4 °C to induce hypothermia; signs of having reached stage III of anesthesia were monitored when loss of balance, loss of operculum movements and loss of reactivity were observed [60]. Later, the sacrificing of the fish was performed using a scalpel by making a cross-section cut in the back of the fish (tail) to obtain blood, which was used immediately to carry out the various analyses.

#### 4.3.6. Analysis of Blood Glucose, Triglycerides, and Total Cholesterol Levels

To measure the different biochemical parameters, the fish in each batch were fasted for 12 h. These were subsequently transferred to fish tanks with glucose-free water for a period of 15 min. Next, the fish in each batch were anesthetized and sacrificed (as shown in the anesthesia and sacrifice section) to obtain blood, which was used to determine the different parameters (glucose, triglycerides, and cholesterol). Glucose levels were measured by means of a glucometer (Accu-Chek, Mannheim, Germany). An Accutrend Plus (Roche, Mannheim, Germany) monitor was used to measure total cholesterol and serum triglycerides, following each manufacturer’s instructions [39].

#### 4.3.7. Evaluation of the Inhibition of Advanced Glycation End Products

The euthanized fish in each group had their eyes removed and a phosphate buffer pH 7.0 was added; the eyes were then macerated followed by centrifugation at 9000 rpm for 15 min. At the end of this period, the supernatant was placed in a 96-well plate for subsequent reading on a fluorometer at an excitation wavelength of 355 nm and an emission of 460 nm.

#### 4.3.8. Statistical Analysis

All data are expressed as mean ± SE. One-way ANOVA analysis. Values of *p* ≤ 0.05 were considered statistically significant. Statistical analysis was performed using the GraphPad Prism 9 software (GraphPad Software Inc., San Diego, CA, USA), version 9.0.2. The experiments were performed in triplicate.

## 5. Conclusions

The methanol extract from *S. purpurea* (CSM) seeds is effective in reducing blood glucose, hypercholesterolemia, and advanced glycation end products. Therefore, this study shows the therapeutic potential of CSM, which can be useful in preventing or delaying some of the symptoms associated with diabetes. As no toxic effects are found, CSM can be used as a safe food supplement with antidiabetic potential, therefore the characterization of its main bioactive components is in progress. It should be noted that the use of zebrafish as a pharmacological model promotes the development of new and effective therapeutic alternatives, as they are used as an initial experimental stage to test new compounds and to study different human diseases without replacing traditional mammalian models.

## Data Availability

The data presented in this study are available upon request from the corresponding author.
